# Effects of Tolvaptan in patients with acute heart failure: a systematic review and meta-analysis

**DOI:** 10.1186/s12872-017-0598-y

**Published:** 2017-06-20

**Authors:** Chunbin Wang, Bo Xiong, Lin Cai

**Affiliations:** Department of Cardiology, The Third People’s Hospital of Chengdu,The Second Affiliated Chengdu Clinical College of Chongqing Medical University, Chengdu, 610031 Sichuan China

**Keywords:** Heart failure, Acute, Tolvaptan, Therapy, Meta-analysis

## Abstract

**Background:**

Acute heart failure, which requires urgent evaluation and treatment, is a leading cause for admission to the emergency department. The aim of this meta-analysis was to evaluate the effects of tolvaptan on acute heart failure and compare them with the effects of conventional therapy or placebo.

**Methods:**

The electronic databases PubMed, EMBASE, and the Cochrane Controlled Trial registry were searched from their starting dates to October 24, 2016. Two authors independently read the trials and extracted related information from the included studies. We used fixed-effects or random-effects models to assess the overall combined risk estimates according to I^2^ statistics. Analysis to determine sensitivity and publication bias was conducted.

**Results:**

Six randomised controlled trials from eight articles, with a total of 746 patients, were included for analysis. Compared with the control, tolvaptan reduced body weight in two days (WMD 1.35; 95% CI 0.75 to 1.96), elevated sodium level in two days (WMD 2.33; 95% CI 1.08 to 3.57) and five days (WMD 1.57; 95% CI 0.04 to 3.09), and ameliorated symptoms of dyspnoea (RR 0.82; 95% CI 0.71–0.95). However, tolvaptan did not improve long-term (RR 1.04; 95% CI 0.66–1.62) or short-term all-cause mortality (RR 0.89; 95% CI 0.45–1.76), incidence of clinical events (worsening heart failure, RR 0.75; 95% CI 0.50–1.12 and worsening renal function, RR 0.97; 95% CI 0.75–1.27), and length of hospital stay in patients (WMD 0.14; 95% CI -0.29 to 2.38) with acute heart failure.

**Conclusion:**

Tolvaptan can decrease body weight, increase serum sodium level, and ameliorate some of the congestion symptoms in patients with acute heart failure, which may help avoid the overdose of loop diuretics, especially in patients with renal dysfunction.

**Electronic supplementary material:**

The online version of this article (doi:10.1186/s12872-017-0598-y) contains supplementary material, which is available to authorized users.

## Background

Heart failure (HF), characterised by volume overload in both extravascular and intravascular spaces, is a common clinical syndrome. It is one of leading causes of admission to the emergency departments, distressing approximately 1–2% of adult population in developed countries [1]. Acute heart failure (AHF) refers to the exacerbation of HF, requiring urgent hospital admission for evaluation and treatment. Although advances in medicine and devices for AHF treatment have reduced AHF-related mortality, hospitalisation rates remain high. There are nearly six million HF patients in America, contributing to one million emergency department visits and over one million hospitalisations annually [2, 3]. Congestion is the major reason for hospitalisation of patients with AHF [4], and severe congestion means poor prognosis [5]. Even with the use of diuretics and vasodilators, congestion persists in many AHF patients, and it has been related to increased morbidity and mortality [6]. Loop diuretics is the first-line therapy for AHF despite serious adverse effects such as decrease in renal function and activation of the sympathetic nervous system and renin–angiotensin–aldosterone system [7]. Additionally, loop diuretics are known to be associated with hyponatremia, renal dysfunction, and hypotension due to loss of intravascular volume owing to sodium depletion [8].

Tolvaptan (TLV), an oral vasopressin V_2_-receptor antagonist, results in the clearance of free water. Previous studies have shown that TLV is a safe and effective drug for the treatment of euvolemic and hypervolemic hyponatremia as well as for patients with HF [9, 10]. Three meta-analyses were performed to investigate the efficacy and safety of TLV treatment in patients hospitalised for HF [11–13]; however, these analyses were primarily focused on chronic heart failure (CHF). Since the publication of these meta-analyses, many new clinical trials have been performed to investigate the efficacy and safety of TVL in patients with AHF [8, 14, 15]. To assess the effects of TLV on AHF completely, we conducted a meta-analysis of randomised controlled trials (RCTs) focusing on the effects of TLV in patients with AHF in comparison with the effects of conventional therapy or placebo.

## Methods

This meta-analysis was conducted in compliance with the Preferred Reporting Items for Systematic Reviews and Meta-Analysis (PRISMA) statement [16].

### Search strategy

We searched the databases of PubMed, EMBASE, and the Cochrane Controlled Trial registry from their starting dates to October 24, 2016 using the following keywords: acute decompensated heart failure OR acute heart failure AND tolvaptan OR vasopressin V_2_-receptor blocker. Our article search was restricted to studies involving human subjects and those published in English. The full search strategies for PubMed are provided in Additional file 1.

### Study selection

We first excluded the reduplicated studies using Endnote software, and then screened the studies according to the titles or abstracts. Two authors (Wang Chunbin and Xiong Bo) scanned the titles and abstracts of all retrieved articles independently, and irrelevant studies were excluded at this stage. The eligibility of the remaining articles was further evaluated for disagreement or uncertainty. Disagreements were resolved by discussion or consensus of a third reviewer.

### Inclusion criteria

The inclusion criteria for the studies were as follows: it should (1) be a randomised, controlled trial (RCT); (2) include participants who are adult patients with AHF, defined as patients had dyspnea at rest requiring urgent hospital admission for evaluation and treatment;(3) compare TLV with controls or other diuretic agents; and (4) include any relevant outcomes: all-cause mortality, clinical events, sodium level, dyspnoea improvement, body weight reduction, and fluid loss.

### Exclusion criteria

The exclusion criteria were as follows: (1) observational study and (2) study on CHF or not reporting the desired outcome.

### Data extraction

Data extraction was performed independently by two authors (Wang Chunbin and Xiong Bo), and the data were checked by a third reviewer. Any disagreements were settled by discussion.

The following information was extracted from each retrieved article: characteristics of included studies (title, first author, publication year, journal, corresponding address, study design, inclusion and exclusion criteria, dose of TLV, treatment duration, and pertinent outcomes).

### Assessment of risk of bias

Risk of bias for included studies was independently assessed by two reviewers by the Cochrane risk of bias tool [17]. Disagreements were resolved by discussion.

### Statistical analysis

We used Stata 12.0 (Stata Statistical Software: Release 12. StataCorp LP, College Station, TX) and Revman software (version 5.3, Cochrane Collaboration, Oxford, United Kingdom) for analyses. Heterogeneity was evaluated by I^2^ test (I^2^ > 50% indicating significant heterogeneity). If there was no significant heterogeneity among the included studies, inverse variance (IV) fixed-effect model was utilised; otherwise, random-effects model was used. In addition, sensitivity analysis was performed to identify the stability of statistical data, and publication bias was evaluated by funnel plots. *P* < 0.05 was considered statistically significant.

## Results

**Fig. 1:**
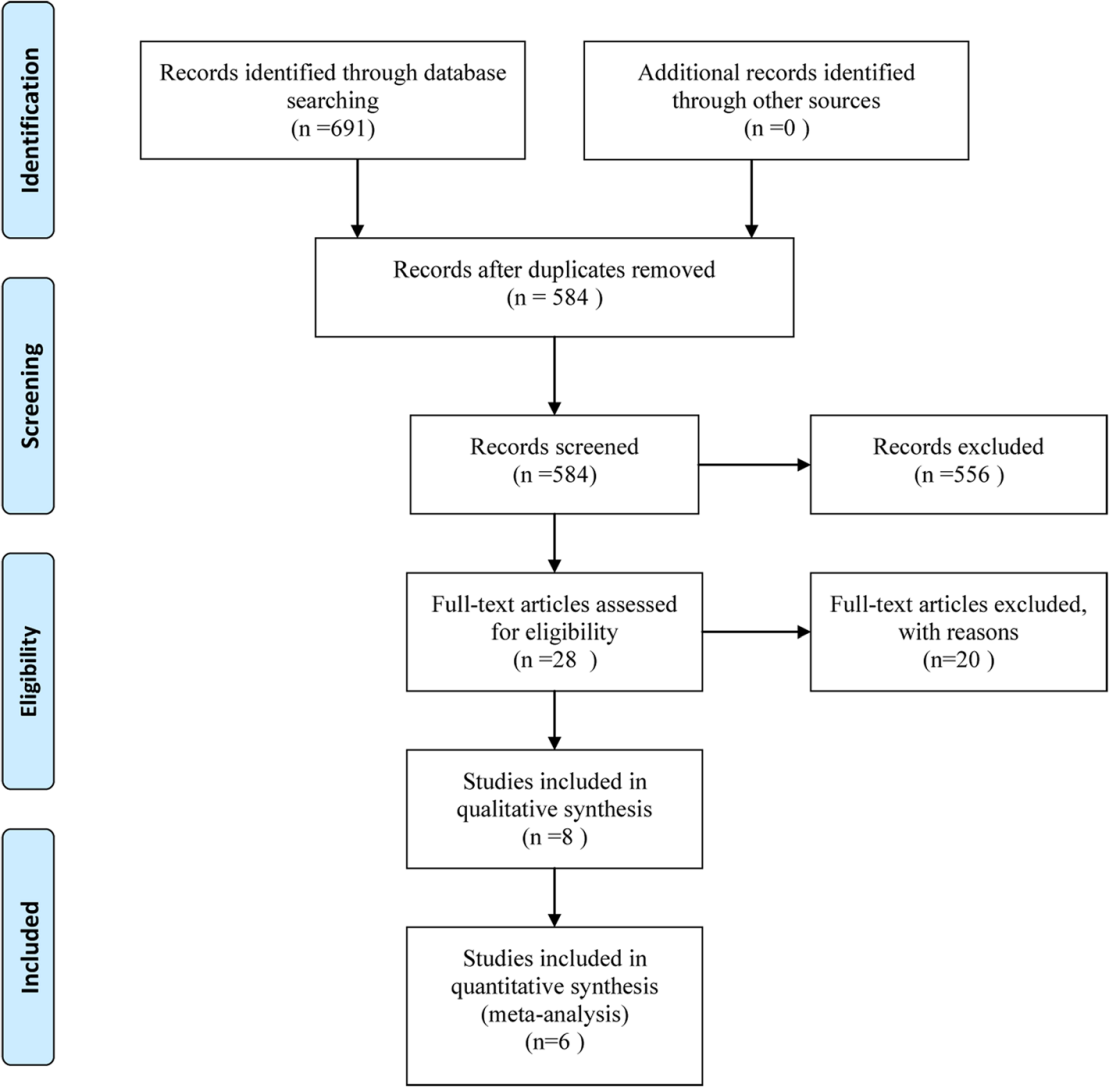
PRISMA flow diagram of study selection

Six-hundred ninety-one articles were identified from the database research: 226 of PubMed, 417 of EMBASE, and 48 of the Cochrane Library. After screening the titles and abstracts, 28 studies eligible for full text screening were identified. A full-text evaluation was performed and 20 were excluded for the following reasons: data published as reviews (*n* = 2), meta-analyses (*n* = 3), study performed on CHF patients (*n* = 5), and failure to report required endpoints (*n* = 10). Finally, six RCTs among eight articles were included. The flow diagram of study selection is shown in Fig. 1.

### Study characteristics and quality

**Fig. 2:**
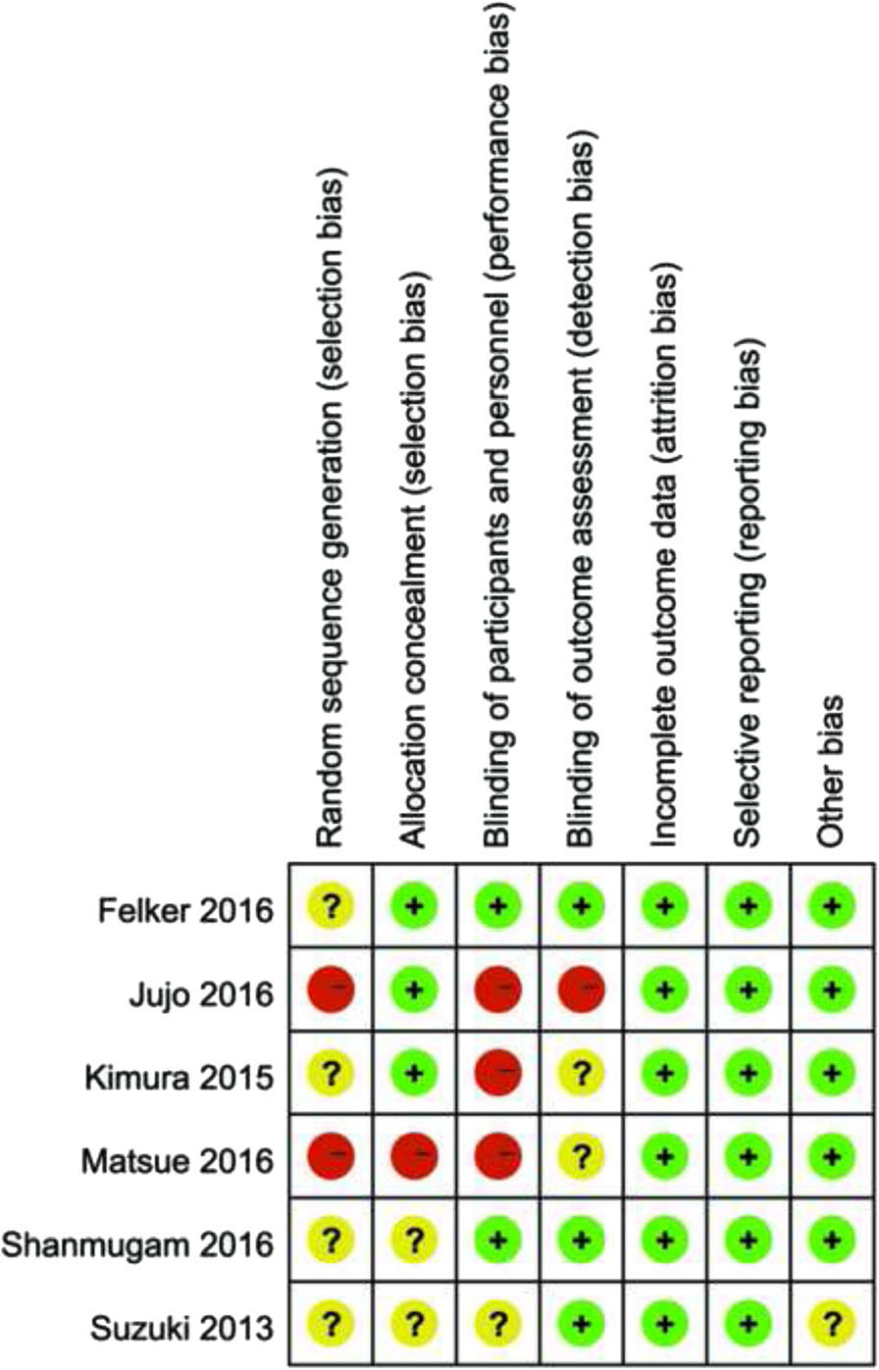
Risk of bias summary

The study characteristics of the six RCTs in the USA, South America, Europe, and Japan from 2012 to 2016, recruiting 746 patients, are presented in Table 1. The mean follow-up duration of the studies was 2 to 636 days. The dose of TLV ranged from 3.75 to 30 mg/day. Two of the studies used placebo as control treatment, three used carperitide or furosemide, and one study compared TLV treatment with conventional therapy. Most participants had AHF [Left ventricular ejection fraction (LVEF) < 50%] of New York Heart Association (NYHA) class II-IV. The risk of bias was evaluated with the Cochrane risk of bias tool. Most items for all included RCTs showed low risk; however, there was insufficient information in some studies, which made the evaluation difficult. Overall, the RCTs included in our meta-analysis were of relatively high quality, except one study by Matsue et al, which showed a high risk of bias [14]. The results are summarised in Fig. 2.

**Table 1:** Baseline characteristics of the studies included in meta-analysis

Study	Subjects	Location	Number (TLV/Control)	Age	LVEF (%) in TLV group/ control group (mean ± SD)	Control	Mean follow-up	TLV dose (mg)/duration (day)	Main outcomes
Suzuki 2013	AHF or acute exacerbation of chronic heart failure, NYHA II-IV	Japan	54/55	74/75	47 ± 18/44 ± 14	carperitide	14 days	3.75–15 mg/d	Symptoms, plasma BNP level, urine volume, adverse events
Suzuki 2014	AHF or acute exacerbation of chronic heart failure, NYHAII-IV	Japan	54/55	74/75	47 ± 18/44 ± 14	carperitide	296 days	3.75–15 mg/d	Serum sodium and potassium, plasma BNP levels, all-cause deaths
Kimura 2015	ADHF	Japan	26/26	80.54/ 86.15	47.54 ± 16.75/ 56.73 ± 11.52	furosemide	7 days	20 mg/d	WRF
Matsue 2016	AHF patients with renal dysfunction	Japan, America	108/109	72.99/ 72.95	45.4 ± 18.1/ 46.8 ± 16.4	no	2 days	15 mg/d	48-h urine volume, WRF, net fluid loss, change in BNP, change in body weight, in-hospital death
Matsue 2016	AHF patients with renal dysfunction	Japan, America	108/109	72.99/ 72.95	45.4 ± 18.1/ 46.8 ± 16.4	no	636 days	15 mg/d	All-cause death
Felker 2016	AHF	America	129/128	66/63	34 ± 17/32 ± 17	placebo	2 days	30 mg/d	Symptomatic endpoints, decongestion and renal endpoints, clinical events
Shanmugam 2016	AHF and concomitant hyponatremia	India	25/26	58.9/57	31.9 ± 12.2/ 29.2 ± 8.7	placebo	5 days	15 mg/d	Sodium concentration, Likert score, adverse effects
Jujo 2016	AHF	Japan	30/30	79/79	NA	furosemide	5 days	7.5 mg/d	Urine volume, BNP, body weight, WHF

*Abbreviations*: *AHF* acute heart failure, *NYHA* New York Heart Association, *BNP* brain natriuretic peptide, *WRF* worsening renal function, *WHF* worsening heart failure

### Effect of TLV on all-cause mortality and length of hospital stay

**Fig. 3:**
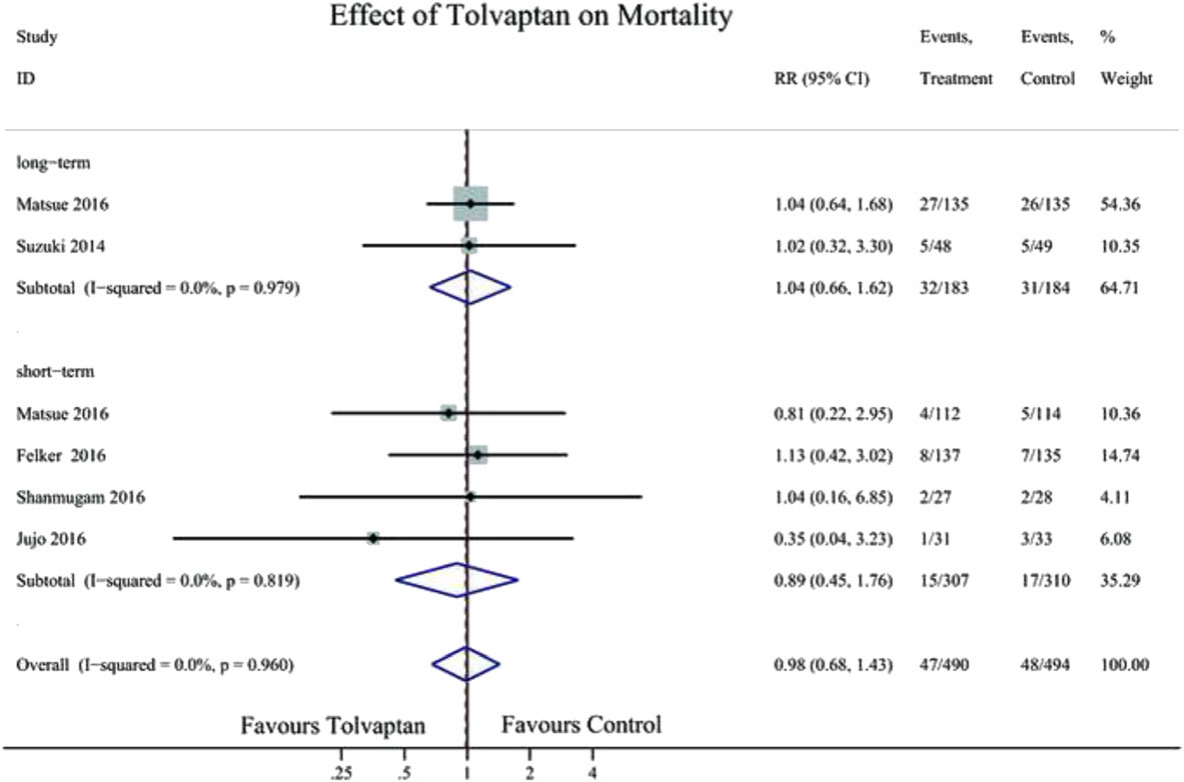
Forest plot depicting the effect of tolvaptan on mortality and clinical events versus control. RR, rate ratio; CI, confidence interval

Six studies reported all-cause mortality of AHF after TLV therapy, two reported short-term (≤30 days) and three reported long-term (>30 days) all-cause mortality. Compared with the control, TLV had no impact on long-term (RR 1.04; 95% CI 0.66–1.62) or short-term all-cause mortality (RR 0.89; 95% CI 0.45–1.76) in patients with AHF (Fig. 3).

**Fig. 4:**
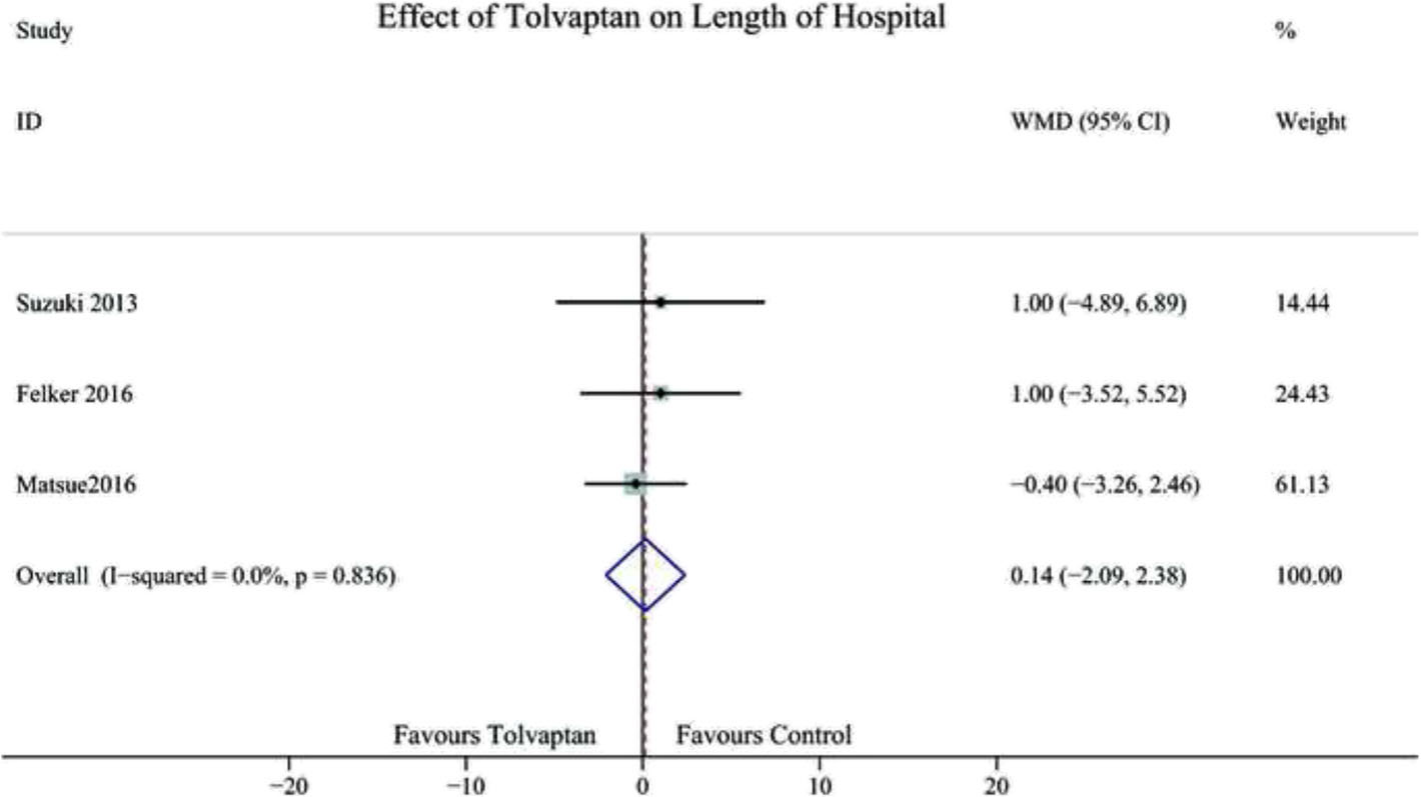
Forest plot depicting the effect of tolvaptan on the length of hospital stay,WMD, weight mean difference; CI, confidence interval

Three studies reported the effect of TLV on the length of hospital stay. The meta-analysis indicated that TLV treatment had no effect on the length of hospital stay (WMD 0.14; 95% CI -0.29 to 2.38) (Fig. 4).

### Effect of TLV on clinical events

**Fig. 5:**
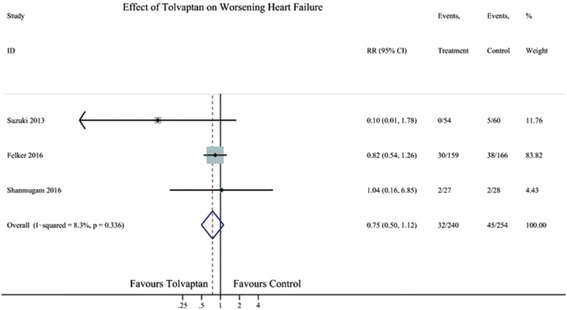
Forest plot depicting the effect of tolvaptan on worsening heart failure versus control. RR, rate ratio; CI, confidence interval

**Fig. 6:**
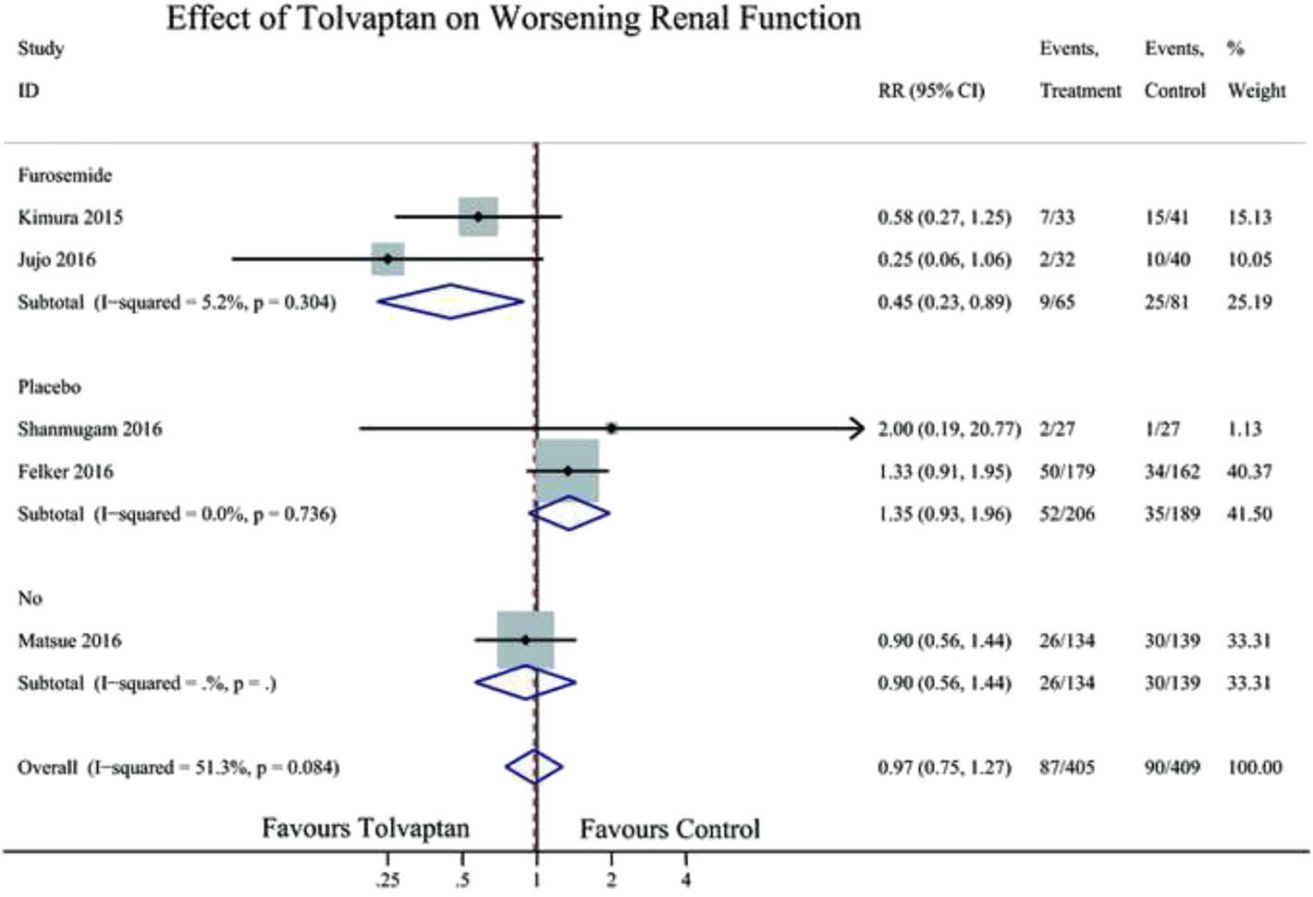
Forest plot depicting the effect of tolvaptan on worsening renal function versus control. RR, rate ratio; CI, confidence interval

Six RCTs reported the effect of TLV on clinical events, three on worsening heart failure (WHF), and four on worsening renal function (WRF). Compared with the control treatments, TLV was not likely to reduce the clinical events of WHF (RR 0.75; 95% CI 0.50–1.12) (Fig. 5) or WRF (RR 0.97; 95% CI 0.75–1.27; Fig. 6).

In subgroup analysis, the control in two study (Kimura 2015, Jujo 2016) were furosemide, in other two study were placebo (Felker 2016,Shanmugam 2016) and no drug in one study (Matsue 2016). In the placebo group, TLV had no effect on WRF (RR 1.35, 95% CL 0.93–1.96), but in the furosemide group, TLV decreased the rate of WRF (RR 0.45,0.23–0.89) (Fig. 6).

### Effect of TLV on dyspnoea improvement

**Fig. 7:**
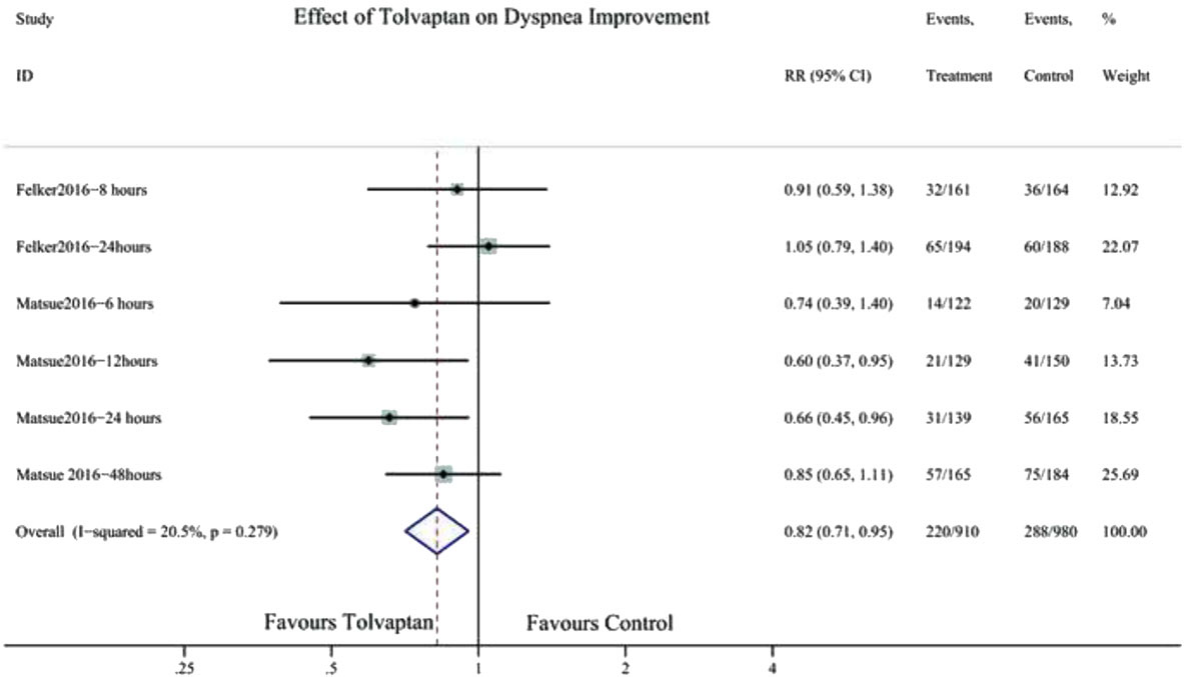
Forest plot depicting the effect of tolvaptan on dyspnoea improvement versus control. RR, rate ratio; CI, confidence interval

Only two studies demonstrated the effect of TLV on dyspnoea at 6, 8, 12, 24, and 48 h. The pooled result showed statistical significance (RR 0.82; 95% CI 0.71–0.95) (Fig. 7).

### Effect of TLV on mean body weight reductions and fluid loss

**Fig. 8:**
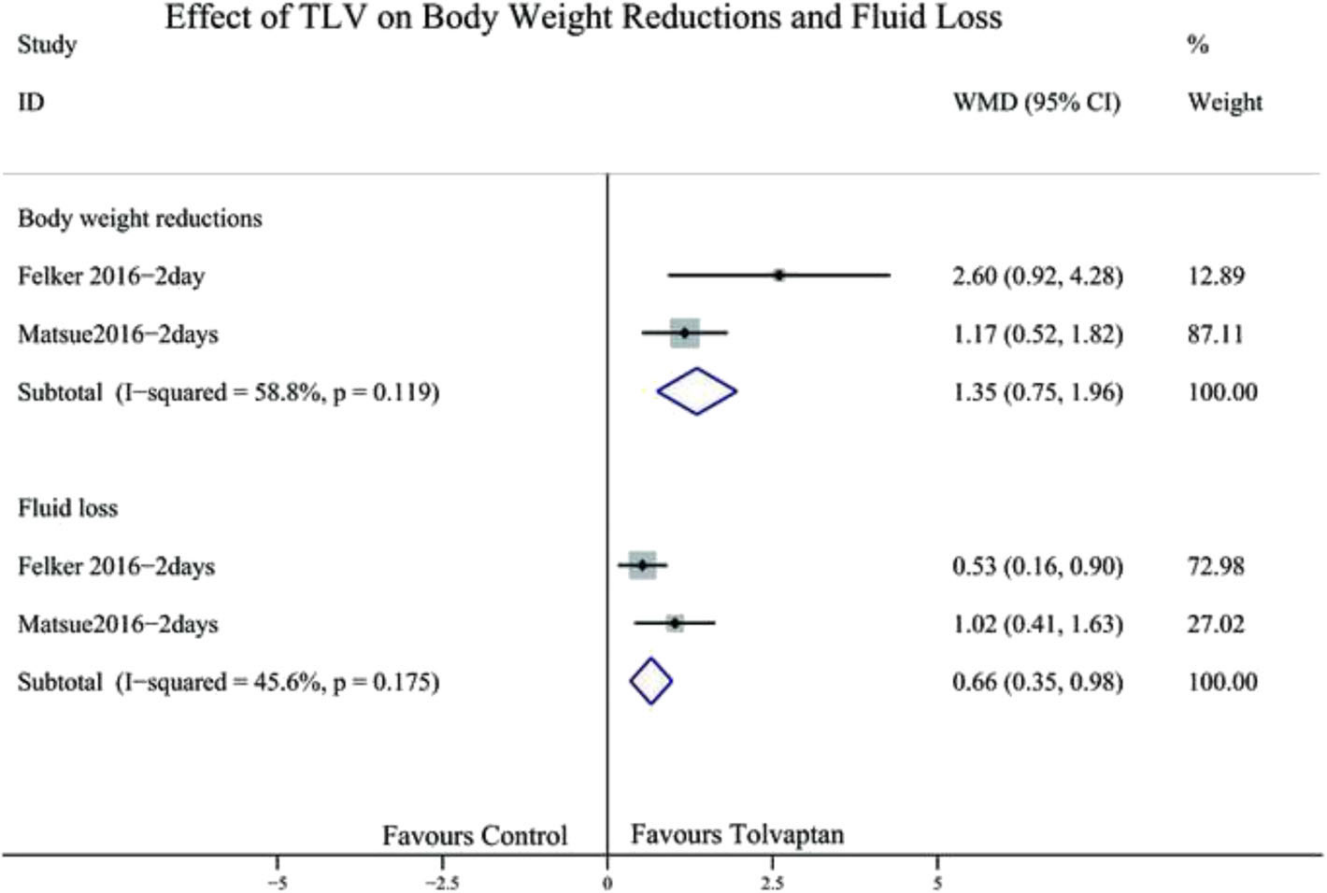
Forest plot depicting the effect of tolvaptan on body weight reductions and fluid loss versus control. WMD, weight mean difference; CI, confidence interval

Mean body weight and fluid loss reflected the aquaretic effect of TLV in AHF patients. In our analysis, TLV could significantly lower the mean body weight two days (WMD 1.35; 95% CI 0.75 to 1.96; Fig. 8). The analysis of fluid loss in two days also showed statistical significance (WMD 0.66; 95% CI 0.35 to 0.98, Fig. 8).

### Effect of TLV on sodium level

**Fig. 9:**
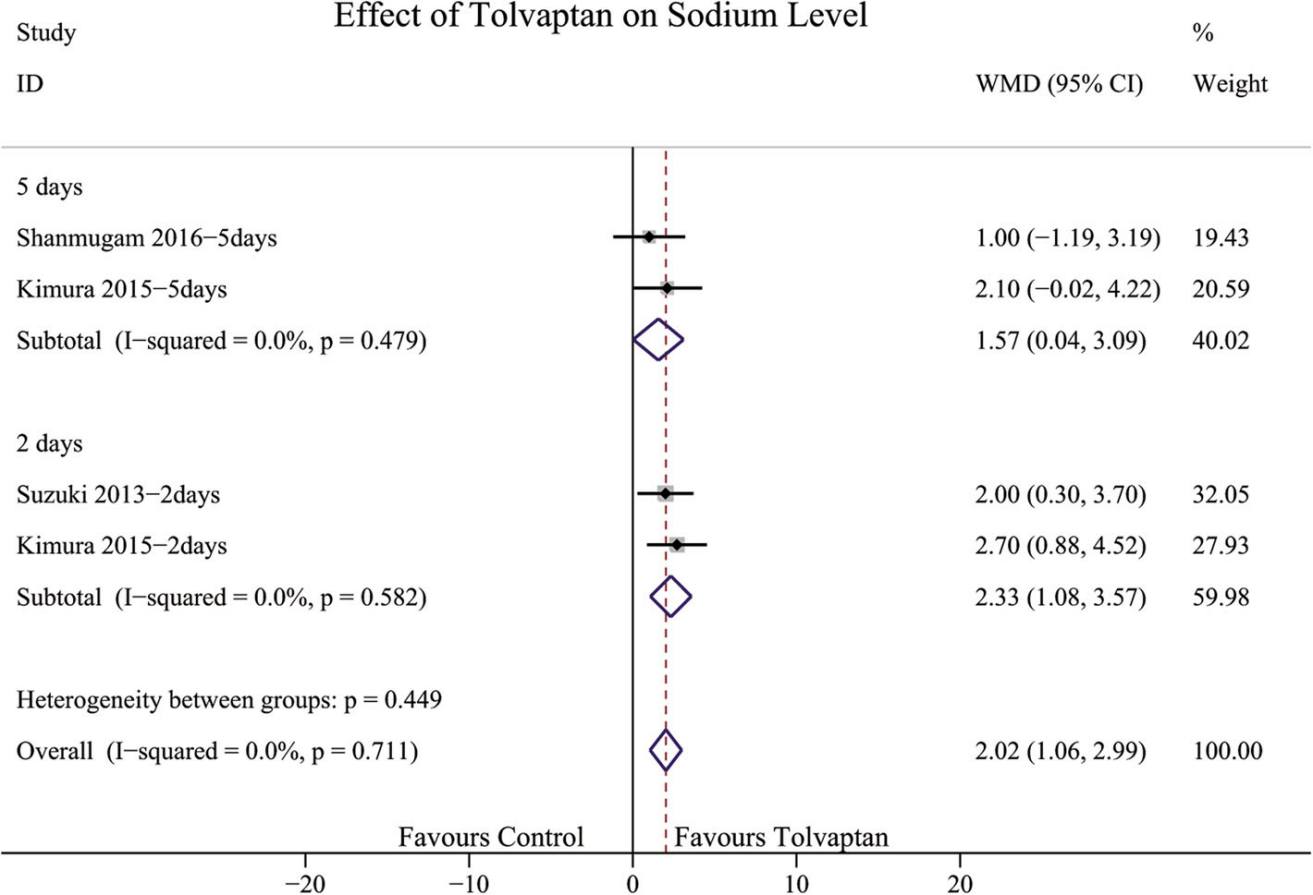
Forest plot depicting the effect of tolvaptan on sodium level versus control. WMD, weight mean difference; CI, confidence interval

Three studies reported the effect of TLV on sodium level at one, two, three, and five days. The results showed statistical significance at two days (WMD 2.33; 95% CI 1.08 to 3.57; Fig. 9) and five days (WMD 1.57; 95% CI 0.04 to 3.09; Fig. 9).

**Fig. 10:**
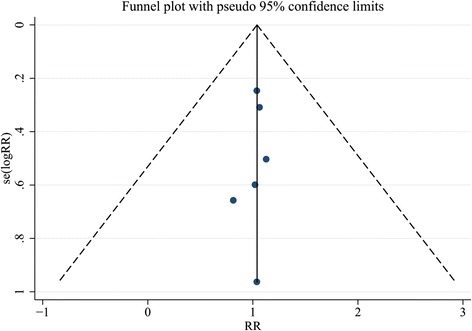
Funnel plot for analysis of all-cause mortality with pseudo 95% confidence limits

**Fig. 11:**
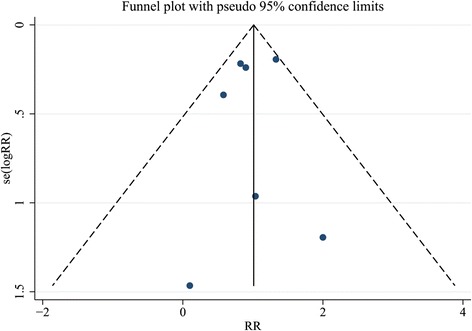
Funnel plot for analysis of clinical events with pseudo 95% confidence limits

Publication bias was not observed for all outcomes in the funnel plots for the analysis of mortality (Fig.10) and clinical events (Fig. 11). Sensitive analyses were performed to investigate the influence of a single study on the overall risk estimate and test the stability of the study. The results did not show substantial difference.

## Discussion

The main findings of this meta-analysis indicate that TLV could reduce body weight, elevate sodium level, and ameliorate symptoms of dyspnoea in patients with AHF. However, TLV did not ameliorate or worsen all-cause mortality, incidence of clinical events (WHF, WRF), and length of hospital stay in patients with AHF.

Congestion is the main reason for hospitalisation of patients with AHF, and decongestion is essential for treating patients with AHF. Loop diuretics are routinely used for natriuresis,timely administration of intravenous loop diuretics is able to save the patient’s life by efficaciously reducing the clinical congestion and immediately decreasing the hemodynamic overload. But high dose of loop diuretics related to renal function deterioration and long-term adverse clinical outcomes [18–20].

TLV, which induces aquaresis, is also effective for decongestion. A retrospective study of 102 consecutive patients with decompensated HF, who were treated with TLV, showed that the early use of TLV reduced the length of hospital stay and mortality [21]. However, in our analysis, all-cause mortality and length of hospital stay had no statistical significance; the mean body weight decreased and sodium concentration increased. To date, only chronic therapy with neurohormonal antagonists, such as β-blockers, angiotensin converting enzyme inhibitors, and angiotensin II receptor blockades, improved clinical outcomes in patients with HF. The results of our meta-analysis showed that TLV as an adjunctive therapy in clinical setting might improve the acute symptoms of dyspnoea; however, it may have no effect on the length of hospital stay or post-discharge outcomes. Hence, the development of better strategies for the treatment of AHF remains a challenge.

Our meta-analysis suggested that TLV could elevate the serum sodium level in patients with AHF in two days. Studies have also shown that decreased sodium is a critical predictor of survival in patients with HF [22]. Being a non-peptide vasopressin type-2 receptor antagonist, TLV is now available for patients with HF having hyponatremia. The study by Shanmugam et al. [8] revealed that TLV at a dose of 15 mg/day is effective in reversing hyponatremia, when administered over a period of five days, indicating that TLV was more suitable for AHF with hyponatremia.

In our analysis, TLV had no effect on the clinical events, WHF and WRF. WHF was defined as worsening of signs or symptoms of HF necessitating an increase in HF treatment. In-hospital WHF could lead to a poor prognosis for AHF patients—both in terms of rehospitalisation and mortality [23, 24]. In the TACTICS-HF study, 90% WHF was treated with additional loop diuretics, and it has been related to a near doubling of post-discharge event rates [23]. Therefore, the use of TLV in AHF may avoid the need for additional loop diuretics and reduce post-discharge event rates.

Renal dysfunction is also a common comorbidity in AHF patients, and it forebodes higher rates of mortality and hospitalisation in patients with AHF to a great extent [25]. A previous meta-analysis showed that TLV elevated the serum creatinine level slightly [11]; however, there was no significant difference in morbidity associated with renal dysfunction between TLV and control groups in EVEREST study [26]. The changes in creatinine levels that occur during successful decongestion therapy do not necessarily indicate the same adverse prognosis [27]. However, renal impairment and WRF, defined as an increase in serum creatinine of 0.3 mg/dL from baseline within 7 days from admission, are often induced by the overuse of loop diuretics [28]. They are associated with increased morbidity and mortality. Renal protective treatment could greatly improve the prognosis of HF patients [29]. In a previous small retrospective study, addition of TLV resulted in more urine output and less WRF compared with that of furosemide [30]. The AQUAMARINE study showed that treatment with TLV resulted in more urine output and dyspnoea relief compared to that with conventional therapy; however, there was no significant difference in the rate of WRF between the groups [14]. In our analysis, although TLV had no effect on WRF overall, while in the subgroup of furosemide, TLV decreased the rate of WRF. The results indicated that use of TLV in AHF might reduce WRF compared with the administration of loop diuretics.

## Limitations

Potential limitations of our meta-analysis should be considered. First, limited number of RCTs was included in our meta-analysis. Only two studies from the selected trials measured long-term mortality and four studies had the outcome of short-term mortality. Second, the duration of TLV use and follow-up time were different in each included study, and this might affect the clinical outcomes. Third, differences in race, age, and complication among studies may result in slightly diverse response to therapy. Fourth, different control treatments might also lead to inaccurate results.

## Conclusions

We observed that TLV did not reduce all-cause mortality, length of hospital stay, and clinical events of AHF. However, the use of TLV could decrease body weight, increase serum sodium level, and ameliorate some of the congestion symptoms, which may avoid the disadvantages of loop diuretics, especially in patients with renal dysfunction. Overall, TLV could be a selective treatment for AHF.

## Additional file

Additional file 1: Search Strategy in PubMed. (DOCX 15 kb)

## Abbreviations

ADHF: Acute decompensated heart failure
AHF: Acute heart failure
BNP: B-type natriuretic peptide
CHF: Chronic heart failure
LVEF: Left ventricular ejection fraction
NA: Not available
NYHA: New York Heart Association
RCT: Randomisedcontrolled trial
RR: Relative risk
TVL: Tolvaptan
WHF: Worsening heart failure
WMD: Weighted mean difference
WRF: Worsening renal function
