# Supplementary material for: Effects of Tolvaptan in patients with acute heart failure: a systematic review and meta-analysis
Source: BMC Cardiovasc Disord. 2017 Jun 20;17:164. doi: 10.1186/s12872-017-0598-y (PMC5479045; doi:10.1186/s12872-017-0598-y)
Supplement: Additional file 1: — Search Strategy in PubMed. (DOCX 15 kb) [file 12872_2017_598_MOESM1_ESM.docx]

Search Strategy in PubMed

(((acute decompensated heart failure) OR acute heart failure) AND Tolvaptan) OR Vasopressin V2 Receptor Blocker(((acute[All Fields] AND decompensated[All Fields] AND ("heart failure"[MeSH Terms] OR ("heart"[All Fields] AND "failure"[All Fields]) OR "heart failure"[All Fields])) OR (acute[All Fields] AND ("heart failure"[MeSH Terms] OR ("heart"[All Fields] AND "failure"[All Fields]) OR "heart failure"[All Fields]))) AND ("tolvaptan"[Supplementary Concept] OR "tolvaptan"[All Fields])) OR (("receptors, vasopressin"[MeSH Terms] OR ("receptors"[All Fields] AND "vasopressin"[All Fields]) OR "vasopressin receptors"[All Fields] OR ("vasopressin"[All Fields] AND "v2"[All Fields] AND "receptor"[All Fields]) OR "vasopressin v2 receptor"[All Fields]) AND Blocker[All Fields])
